# In arms and on slides: slavery-related interests in the dissemination of smallpox vaccine in Brazil and Angola, 1804-1810

**DOI:** 10.1590/S0104-59702026000100027en

**Published:** 2026-08-07

**Authors:** Jaqueline Hasan Brizola, Gutiele Gonçalves dos Santos

**Affiliations:** i Postdoctoral researcher, Graduate Program in the History of Science and Health/Casa de Oswaldo Cruz/Fiocruz. Rio de Janeiro – RJ – Brazil. brizajaque@gmail.com; ii Doctoral student, Graduate Program in the History of Science and Health/Casa de Oswaldo Cruz/Fiocruz. Rio de Janeiro – RJ – Brazil. gutielegoncalves12@gmail.com

**Keywords:** Vaccine, Smallpox, Slavery, Bahia, Angola

## Abstract

This article discusses the intersections between vaccination and slavery in colonial Brazil, highlighting the arrival of the smallpox vaccine in Bahia and its spread to other regions of the Portuguese Empire including its holdings in Africa. We highlight the story of enslaved boys, who were primarily inoculated and vaccinated between the late eighteenth and early nineteenth centuries, through analysis of bibliographical and documentary sources that illustrate the actions of historical figures such as Felisberto Caldeira Brant, Francisco da Cunha Menezes and Francisco Ignácio Sequeira Nobre. These activities reveal the initiative of slave owners in the search for solutions against smallpox, highlighting the complex relationships between slavery, science and health.

In 1805, only a few years after the development of the smallpox vaccine was announced in Europe, debates on how to transport and implement this preventive method were taking place in Brazil and Africa. This article explores the intersections between vaccination and slavery during the first decade of the nineteenth century, focusing on the processes surrounding the vaccine’s arrival in Bahia in 1804 and subsequent shipment of material for smallpox vaccination to other parts of the Portuguese Empire, including Angola. Along with examining aspects related to how the vaccine was received (only via the arms of enslaved and indigenous individuals, in Brazil), we will attempt to depict the activities of traders working in Bahia and bureaucrats in the service of the Portuguese kingdom in Brazil and Angola to distribute the vaccine in colonial territories.

The vaccination technique proposed by Edward Jenner was based on his observation that people who had come into contact with the bovine virus (cowpox) did not contract the human version (smallpox). By inoculating an 8-year-old boy with “bovine pus” and subsequently exposing him to smallpox, Jenner demonstrated the effectiveness of his vaccine, which was named for its origin in cows (*vacca* in Latin) (Chalhoub, 1996, p.136). Knowledge of Jenner’s experiments quickly spread across the world, and the ancient practice of variolation to inoculate healthy people with the human smallpox virus was gradually replaced by vaccination after the introduction of the bovine smallpox virus (Esparza, 2020).^1^

Meanwhile, in the Americas smallpox had wreaked havoc since the first contacts with Europeans in the fifteenth century, and was considered more lethal than gunpowder as a weapon to exterminate the native peoples (Crosby, 2001; Malvido, 2003; Anzolin, 2014).^2^ But in Brazil, new epidemics were commonly blamed on the enslaved people arriving from Africa (Carvalho, 2006). The endemic nature of smallpox on that continent, especially during the eighteenth century, led to the notion that these individuals were responsible for the uncontrolled wave of this disease in Brazil (Alden, Miller, 1987).^3^

Unlike in other countries, where the nobility had their children vaccinated (Duro Torrijos, 2014, p.320), in Brazil the first tests were conducted on enslaved and indigenous people.^4^ Official documents in the National Library of Rio de Janeiro show that Felisberto Caldeira Brant, a merchant from Minas Gerais who worked in Bahia, sent seven boys he owned to Lisbon in 1804 to be inoculated with cowpox (Chalhoub, 1996; Portugal, 2018; Brizola, 2022). When they returned to Brazil, these boys formed the foundation of the country’s first vaccination plan.^5^

In addition to Caldeira Brant, the glass trader Francisco Ignácio de Sequeira Nobre, and the governors of Bahia and Maranhão, Francisco da Cunha Menezes and Antonio de Saldanha da Gama respectively, also served as important interlocutors in the vaccine’s arrival in Brazil and subsequent shipment to Angola. By analyzing the trajectories of these agents, we reveal the conflicts and interests surrounding immunization, the movement and development of individuals around “scientific discoveries” of that time, and socially accepted ways of preventing smallpox, as well as highlight the importance of slavery and slaveholding interests to the history of science in Portugal’s colonial territories during the early nineteenth century.

The time frame we selected for this analysis comprises a transition period with regard to the interpretation of diseases or ways to combat them: during the second half of the eighteenth century, the Enlightenment granted medicine and certified physicians prominent positions in “preventing,” diagnosing and “curing” diseases (Walker, 2013). As for documentation, we must mention the official correspondence between Francisco da Cunha Menezes and João Rodrigues de Sá e Melo, Viscount of Anadia in Lisbon, which clarified aspects related to the vaccine’s introduction in Brazil from 1804 onward. Other documents in the Bahia State Public Archives and the Overseas Historical Archive in Lisbon contributed to our understanding of the processes related to transporting and shipping cowpox to other locations, through either the “arm-to-arm” technique or on glass slides produced to hold the pus used in vaccination (Fernandes, 2003).

The information extracted from the various sources comprised a set of data that was collected and analyzed using qualitative methodology to lend meaning to discourses by agents involved in smallpox vaccination, while not forgetting that these discourses themselves carried certain intentions. Alongside analysis of the sources, we also needed to see the processes involving the smallpox vaccine through a social history lens, while also dialoging with contributions from postcolonial theory. According to Bhabha’s (1998, p.239) definition, the postcolonial perspective is rooted in the testimony of countries considered peripheral, where contributions by “minorities” are valued within the networks of relationships that form between what came to be called the center and the periphery.

References to Bahia as a vaccine distribution center lead us to reconsider the role of agents who worked here, who were not only subjected to imperial decisions but also acted to benefit their own businesses and slavery. While traders like Felisberto Caldeira Brant and Francisco Ignácio de Sequeira Nobre were in line with ideas coming out of Portugal, their actions highlight the close relationship between slave interests and the vaccination plan for Brazil and Angola during the early nineteenth century.

Keeping this scenario in mind, this article explores some questions. How did the experience of slavery and slave-related interests influence the development of practices adopted to “prevent” smallpox in colonial territories like Bahia and Angola? What agreements were made in order for the Jennerian vaccine to arrive in Brazil in the early nineteenth century, and who were the agents involved in dissemination? Additionally, how were enslaved bodies used to propagate the vaccine in territories controlled by the Portuguese Empire in the Americas and Africa?

The history of the smallpox vaccine involves multiple variables in the scientific, political and social spheres, but gaps still remain in terms of understanding the impacts that resulted from combating this disease in slaveholding contexts. In this case, the practices surrounding the vaccine in Bahia and Angola offer fertile ground for us to comprehend the connections between health, medicine and slavery. The temporal and geographic issues outlined here make it possible to understand the dynamics related to adopting, encouraging and shipping the Jennerian vaccine across the Atlantic during a period marked by the diffusion of scientific practices to combat contagious diseases.

Studies on epidemics, science and slavery have advanced over the past decades, contributing to historical knowledge and providing explanations on the methods used to combat the epidemics that frequently plagued the Americas since the first contacts between Europeans and the peoples originating in Africa and the Americas (Sheridan, 1985; Alden, Miller, 1987). The debates that emerge in these areas, especially since the 1980s, have incorporated multiple historical experiences recognizing health and disease within a construct and a social framework that takes into account historical time, social groups and the individuals involved (Rosenberg, 1992).

In recent decades, authors such as Derrick Baxby (1996), Donald Hopkins (2002) and Pierre Darmon (1986) have noted that practices similar to smallpox vaccination were already used by rural populations in various places even before Jenner’s vaccine arrived as a preventive method, revealing how these initiatives were appropriated by imperial efforts during the eighteenth and nineteenth centuries.

Recalling the notion of European triumphalism, Michael Bennett (2020, p.6) states that spreading the smallpox vaccination around the globe was a humanitarian effort. By questioning assumptions related to delays and colonial dependence, this author calls attention to the role of slavery in disseminating the vaccine across the Americas, since most of the vaccine’s international trajectory during the early nineteenth century involved slave trading posts (Bennett, 2020, p.319). Bennett’s work merits attention for its global analysis of the reality of this vaccine in several countries including Brazil.

In Africa, where smallpox was a permanent disease, this new preventive method meant that the lives of many of the people who were sold as part of the trans-Atlantic slave trade could be saved. The historian Mariana Cândido’s detailed study on the port of Benguela (2013) shows that events in Brazil affected the African coast politically as well as socially, with the slave trade linking Brazil and Africa in aspects extending beyond trade. Cândido, who stressed the importance of the South Atlantic as a space for the circulation of people, ideas and cultures, did not fail to recognize the severity of smallpox epidemics, which recurred in Benguela throughout the nineteenth century.

For this reason, according to Jill Dias (1981, p.364) the more powerful members of the colonial community acted quickly to avoid the death of their captives. As this author pointed out, as early as the 1860s the entrepreneur João Caetano Sarmento, who was active in Angola, had all of the enslaved people he owned in Luanda, Benguela Velha and Dombe Grande vaccinated at the beginning of a smallpox epidemic (Dias, 1981, p.364). In this case, the vaccination of African slaves, which was initially supported by traders from the captaincy of Bahia, was continued and also adopted by local slave owners in different regions of Angola.

Joseph Miller (1988, p.213) stated that new medical technologies recognized as effective against tropical diseases were gradually incorporated into the culture of many Africans living on the west coast of the continent, especially in Angola, where the main ports (Luanda and Benguela) had direct contact with travelers from the Americas and Europe. But this author argued that the Jennerian vaccine only reached Angola in the 1820s, when it significantly reduced annual variation in mortality among enslaved people (Miller, 1988, p.436).

In Brazil, after the pioneering work by Sidney Chalhoub (1996) and Tania Fernandes (2003) that analyzed social and institutional aspects of variolation and vaccination practices, a new generation of historians examined the issue of the smallpox vaccine and its impact on controlling this disease (Pimenta, Gomes, Kodama, 2018; Portugal, 2018; Brizola, 2022; Santos, 10 maio 2024). Fillipe Portugal (2018), who studied the introduction of vaccination within the Portuguese Empire based on cases from Brazil and Portugal, highlighted the pioneering spirit of Bahia, Rio de Janeiro and São Paulo as they implemented the cowpox vaccine from 1803. This author did not ignore the role played by slave traders from Bahia during the early days of vaccination in Brazil, examining the work of pro-vaccine physicians such as José Avelino Barbosa in Salvador and Hercules Otaviano Muzzi in Rio de Janeiro.

Regarding the vaccine’s arrival in the province of Bahia and the city of Salvador, Hochman and Souza (2022) maintain that these sites served as “innovation centers” in terms of the public response to smallpox. By analyzing the perceptions and actions of public authorities in the process of producing and preserving smallpox pus for vaccination, these authors make an important contribution to understanding the role of Bahia as a center for distributing and administering vaccines during the first decade of the nineteenth century. Hochman and Souza (2022, p.3432) also mention that vaccines were shipped from Bahia to Angola, according to an 1811 report in the *Gazeta do Rio de Janeiro*.

Bahia’s pioneering role as an early vaccine distribution center has also been highlighted by historian Maria Gabriela Marinho (2015, p.155). This author analyzed the circuit of communications related to smallpox vaccination between the royal court and governors of the overseas territories. In her investigation of documentation in the Overseas Historical Archive, Marinho concluded that a kind of colonial sanitary administration existed from the late eighteenth century to the early nineteenth century, which was able to guide and coordinate initially variolation with variolic pus, and later vaccination with cowpox*.* Marinho suggests that Bahia may have assumed some relevance as a “depot” for redistributing the vaccine and edicts issued by the royal court.

In agreement with Marinho and Hochman and Souza on Bahia’s groundbreaking position vis-a-vis vaccine distribution processes among the Brazilian captaincies, we highlight the interlocutions between Brazil and Africa during the process of disseminating the smallpox vaccine through the “arm-to-arm” method or transport of variolic pus in glass slides.

As we shall see, the networks of relationships that permitted the early diffusion of immunization involved merchants, doctors, and Portuguese bureaucrats. Analysis of these subjects and their actions is representative of the fight against a disease that caused enormous damage throughout history, but also demonstrates the interest of slave-holders that mediated these activities in terms of “preventing” smallpox. Adaptation to the colonial context led to unique ways of applying science, since the first experiments with the vaccine in Brazil were only possible through the use of indigenous and enslaved bodies.

## Circulation of knowledge and slaveholding interests in the dissemination and transport of the vaccine

The vaccine reached Brazil at the same time it was introduced in Portugal.^6^ Following a trend seen in other kingdoms, in an attempt to legitimize the new technique Prince Regent João VI had his children vaccinated in 1804, and other Portuguese nobles soon followed suit. In this way, the animal-based vaccine quickly supplanted inoculation with human smallpox, and the house of Bragança began to carry out an “absolutist policy in favor of vaccination” (Portugal, 2018, p.42).

The vaccine arrived in Bahia in 1804, carried in the bodies of seven enslaved boys. According to Camargo (2008) and Brizola (2022), this was not the first time the Jennerian vaccine was used in Brazil, as there are official records of vaccination in São Paulo from 1803. These documents, preserved in both the São Paulo Municipal Historical Archive and the Overseas Archive in Lisbon, indicate that the immunization initially circulated in São Paulo before formally arriving in Bahia. However, the initiative that took place in Bahia undeniably led to greater prominence for vaccination in Brazil. In 1805, Bahia governor Francisco da Cunha Menezes offered details of how the vaccine arrived, revealing the use of enslaved arms in the process:

Having arrived at this port on this past December 30, the ship Bom Despacho, which carried the seven black boys sent from here by some traders of this city to carry the vaccine, began on the following day to use this prophylaxis against smallpox with administration of the humor from the last of the vaccinated seven [boys] to over one hundred people of varying ages by the ship’s surgeon Manoel Moreira da Rosa and the physician José Avelino Barbosa in my presence (Ofício..., 4 jan. 1805).

According to the governor’s report, the vaccine arrived in 1804 via the “arm-to-arm” method, which consisted of inoculating an individual with fresh fluid from the pustule of another person during the peak of their reaction. Note that by using the bodies of enslaved children to transport the pus used in this process, other people could be vaccinated at that time, and it appears that the expedition accompanied by the physician José Avelino Barbosa and the surgeon Manoel Moreira Rosa succeeded in its initial objectives.

Research in handwritten documents reveals the networks of relations established between agents of the Portuguese Empire who worked in the captaincies of Brazil and groups of individuals interested in vaccination. From a social history viewpoint, this scenario helps us understand how slaveholding interests and race mediated the different relations established during the colonial period, including the most acclaimed scientific discoveries of that era.

In this case, starting in 1804, the processes involving the vaccine obeyed different interests which not only addressed smallpox but also dialogued with the interests of slavery, since the men who dedicated themselves to spreading the vaccine in the early days were rich traders and owners of enslaved people. This tendency has also been observed in other strategic locations in the Americas. The Cuban historian Beldarraín Chaple (2015) noted that after 1804 concerns grew about the vaccination of slaves in Cuba, especially recently arrivals from Africa, since smallpox represented incalculable economic damage to slave traders and owners.

As for the vaccine’s arrival in Bahia, Michael Bennett (2020, p.314) highlights the important role played by the physician José Avelino Barbosa, who was in England in 1803 and obtained samples of the vaccine. According to Bennett, dialogue between Barbosa and Felisberto Caldeira Brant led to organization of the expedition to Lisbon involving the enslaved boys in 1804, with support from the Bahian governor Francisco da Cunha Menezes. Although we cannot disregard Menezes’ role with regard to the transport and arrival of the cowpox vaccine in Brazil, the documentation indicates active participation by other agents.

In this case, individuals directly linked to the slave trade were very important to vaccination in Brazil at the start of the nineteenth century; this runs counter to the notion of a passive colony that only received orders vertically, without any type of negotiation or mediation with the metropolis. The agents involved in vaccination in Brazil had relative autonomy to take the necessary measures for immunization during the early days.

Notably, the notion that Brazil was passive in relation to Portugal under the colonial system has been challenged by Brazilian historiography for over two decades; one milestone in this discussion was the publication of the book *Antigo regime nos trópicos* [Old regime in the tropics] (Fragoso, Bicalho, Gouvêa, 2001). By observing the autonomy of agents involved with the vaccine in Brazil, we are providing an example of what Antonio Manuel Hespanha (1994) called the plurality of political ties in relations between local power and the metropolis, where actions aimed at the interests of the powerful locals were not uncommon regardless of royal consent.

Bahia governor Francisco da Cunha Menezes sent a letter to the court reporting the efforts by the physician José Avelino Barbosa, who was responsible for vaccination in that captaincy. After the initial applications of the vaccine in Bahia, Barbosa appears to have sent his observations to Edward Jenner himself. Addressing the Prince Regent in 1805, Menezes stated:

The physician José Avelino Barbosa, whom His Royal Highness charged with directing vaccination in this captaincy, presents me with these two letters, one to the chief surgeon of the armada Teodoro Ferreira de Aguiar, and the other to Doctor Jenner in London, in which he contributes some observations regarding the vaccine in this country (Ofício..., 26 jan. 1805).

In addition to using enslaved boys for inoculation and for transport of smallpox material between Bahia, Rio de Janeiro and Angola, doctors like José Avelino Barbosa who were in charge of applying the vaccine also reported their experiences in correspondence abroad. As reported in the 1805 letter from the governor of Bahia, Edward Jenner is thought to have received a letter from José Avelino describing the initial experiments with cowpox in Brazil.^7^ We do not know where this correspondence is located or what it contained, but the information provided by Menezes seems to indicate at the very least that the efforts in Brazil and especially in Bahia to apply the vaccine were notable at that time; otherwise, there would be no reason for communication with Jenner in London.

In any case, a report by the governor Francisco da Cunha Menezes himself indicates that 1,300 people were vaccinated in Bahia in 1805, including locations in Bahia’s Reconcavo region, specifically sugar mills (Ofício..., 1 jun. 1805). This last detail indicates that enslaved workers on the sugar plantations in northeastern Brazil were among the priority groups for initial vaccine tests in the captaincy of Bahia, since these locations held dozens or even hundreds of slaves who produced the sugar Brazil exported.

As for transport of the pus used in vaccination, we must highlight Francisco Ignácio de Sequeira Nobre, a rich slave trader who worked in the glass manufacturing sector in the early nineteenth century. His activities related to transporting vaccine pus are closely linked to his trade, since he requested favors in Lisbon in order to expand his business.

In his 1810 text *Observações sobre a franqueza da indústria e estabelecimento de fábricas no Brasil* [Observations on the open nature of industry and establishment of factories in Brazil], José da Silva Lisboa (1999, p.75) mentioned that according to the charter of April 28, 1809, “inventors and introducers of some new machine or invention in the arts” would have the privilege of exclusivity in business for fourteen years, ensuring the owner an “extensive, progressive and profitable market.”

These terms describe Francisco Ignácio de Sequeira Nobre’s business well, since in addition to trading enslaved people to Bahia in the early nineteenth century, he also founded Brazil’s first glass factory in the captaincy in 1810 after receiving royal authorization. The factory produced a wide variety of items ranging from decorative items to medical utensils like medicine bottles and suction cups (Notícia..., 1813).

Investigations of how vaccine pus arrived in Brazil reveals Sequeira Nobre’s role in disseminating smallpox vaccination, since he is mentioned in official documents as responsible for transporting vaccine material, including to Angola to vaccinate enslaved people there. This procedure was intended to minimize the risk of smallpox outbreaks, both during the voyage on the slave ships as well as upon their arrival to the ports in Salvador, where epidemics could spread rapidly between the newcomers and the local population.

Due to the difficulty of transporting people who had been inoculated with the virus and the risks associated with this practice, vaccine material was largely transported in glass slides in Brazil, especially after the Royal Vaccine Board was created in 1811 (Fernandes, 2010). This method used what was known as dry matter and involved placing pus from a patient’s arm between two glass slides, where it quickly dried, facilitating transport and shipment of this material to other regions. For vaccination, this dry material needed to be diluted in a small amount of cold water, stirring it with the tip of a lancet for a few minutes until the mixture turned slightly opaque (Chernoviz, 1851, p.455).

In return for making glass objects that could be used to transport medicines (including the dry matter for vaccination), Sequeira Nobre asked the king for exclusive rights to produce and sell glass in Brazil for a period of twenty years. The monarch did not grant this privilege exactly as requested, but offered some guarantees in recognition of the benefits that his business produced for the kingdom (Carta do príncipe..., 1 set. 1810).

The vaccine-related activities of individuals like Sequeira Nobre and Felisberto Caldeira Brant expose the shifting nature of social relationships involving exchanges or privileges and favors that were typical of colonial societies. To promote vaccination in its overseas colonies, the Crown was aided by individuals who ensured their own privileges by providing services for the common good. In fact, it is important to highlight Lisbon’s role in collaborating to transport and apply vaccination in Brazil as well as in other parts of the Empire (Saavedra, 2004).

But Sequeira Nobre’s interest in disseminating the vaccine notably also had economic motivations. Vaccinating enslaved people in ports before they crossed the Atlantic minimized the risk of outbreaks on board, avoiding major financial losses for the slave traders. And since they were brought to sales warehouses when they arrived, ensuring that the slaves were protected against smallpox was essential for trade. It avoided the spread of epidemics and preserved “public health,” along with the profits of traders involved in the trans-Atlantic slave trade. In this way, the “health” of enslaved people was instrumentalized according to commercial interests, since vaccination practices were linked to the slave system and economic dynamics of the time.

## A shipment to Angola: slave-trade dynamics in the fight against smallpox

As we have seen, the role of the glass trader Francisco Ignácio de Sequeira Nobre in transporting vaccine material extended beyond the captaincies of Rio de Janeiro, São Paulo or Minas Gerais, where he maintained supply stations for his business. As the cowpox vaccine technique was mastered, the governor of Bahia sent the dry matter in glass slides to Brazil’s other captaincies as well as Angola. According to Menezes,

In the report dated March 15 of this year, I informed Your Excellency that I had not only sent vaccine humor in glass to the governors and captains-general of the Kingdom of Angola and São Paulo, but also sent on His Majesty’s [ship] that was carrying tobacco from India to Rio de Janeiro Jorge Francisco Machado, assistant surgeon of the first line regiment of this city, with four boys for vaccinating during the voyage (Carta de Francisco..., 1 jun. 1805).

The glass slides Sequeira Nobre manufactured were one option for shipping vaccine material, including over long distances. But transporting the vaccine in the arms of enslaved boys also took place at the same time, ensuring the circulation of cowpox and its effects in parts of Brazil and Africa as early as the beginning of the nineteenth century.

On the topic of epidemics on the west coast of Africa, Alden and Miller (1987, p.203) presented data proving that smallpox occurred endemically and in the form of epidemics throughout the seventeenth century. This led to frequent complaints by slaveowners, who reported losing their entire workforce to the disease. In response, authorities in Bahia ordered a specific quarantine for enslaved people arriving from Angola, although they were not able to effectively enforce this restriction (Hochman, Souza, 2022, p.3431). Given the difficulty of monitoring and controlling ships as they arrived, investing in vaccination of arriving Africans consequently became the most viable alternative.

Another important player who appears at the center of discussions regarding the vaccine during this period is Antonio de Saldanha da Gama, who served as governor of Maranhão in 1805. He had the confidence of the Crown and was later transferred to work in Angola, where he encouraged “sanitary exchanges” between the colonial territories.^8^ The transfer of governors and trusted men to different locations was common in Portugal’s extensive empire. According to Gouvêa, Frazão and Santos (2004, p.103), some specific details and mechanisms guided social, political and economic practices in colonial Portuguese society; these authors maintain that the multicontinental nature of the Portuguese Empire was one reason many senior officers served in different overseas territories throughout their careers.

This was the case for Saldanha da Gama, who worked to distribute the animal vaccine in Angola after learning about its benefits. Before his arrival, he already had experience with the vaccine when he served as governor of Maranhão. Writing from São Luís in 1805, Saldanha de Gama reported his experience having the vaccine sent from England, and referred to the Bahian governor’s work in favor of the vaccine:

Instigated by his well-known zeal and patriotism he sent a ship [to Maranhão] and inoculated some slaves in the presence of the captain of that ship, teaching and demonstrating the instruments in order to continue this operation, accompanying this shipment with dry matter preserved in glass (Ofício…, 24 jun. 1805).

His report went on to say that when the dry matter sent by the governor of Bahia arrived in Maranhão, it was applied that same day in “slaves who have not yet had smallpox.” However, the “desires of the tireless governor were unsuccessful,” because “the master of the ship either slanderously or through other circumstances claims to have allowed the material to dry out upon passing it on to the bodies” (Ofício, 24 jun. 1805).

This was the second unsuccessful experience with vaccination during Antonio de Saldanha da Gama’s administration. The first attempt was the purchase of “six boys from the city of Bahia, for them to go from Salvador to São Luís over land, accompanied by a skilled surgeon who will bring them to the city with the vaccine pus that can be propagated in the captaincy” (Informação..., 27 jan. 1806).

As we can see, the use of enslaved people involving both the “arm-to-arm” method and glass slides was an integral part of the smallpox vaccination plans, and not only in Bahia. In announcing the purchase of “six boys” to bring vaccination to Maranhão, Saldanha da Gama demonstrated that enslaved boys may have been a recurrent means of transporting the vaccine internally in the early nineteenth century.

In 1806, Saldanha da Gama left Maranhão to become Governor General of Angola. But even during this transition he continued to be active in debates on vaccination. In 1810, in a letter sent when he was in Angola, he reinforced the tireless zeal of Francisco Ignácio de Sequeira Nobre, who sent

a warship to deliver the vaccine pus to that country to avoid the disastrous consequences that smallpox had caused during the previous year. This expedition was promoted by the aforementioned Francisco Ignácio de Sequeira Nobre, as the governor of that captaincy communicated to me and which he did diligently but even at his own expense, for which I address my thanks to him (Gama, 1 set. 1810).

Saldanha da Gama’s letter, however, does not provide details about the transport of vaccine pus. In this case, we cannot determine whether in 1810 the vaccine reached Angola through enslaved boys (repeating the 1804 trip to Bahia) or whether it was transported in glass slides. What we do know, given the governor’s report, is that the inoculant arrived in Angola in the early nineteenth century thanks to the efforts of Sequeira Nobre, owner of Brazil’s first glass factory, which also had links to the lucrative trade in enslaved people between the coasts of Africa and Brazil.

There are few reports on the diffusion of the Jennerian vaccine in Angola during the first half of the nineteenth century. However, Joseph Miller states that Jenner’s technique is thought to have significantly decreased mortality in the individuals trafficked to the Americas from 1820 onward. Miller (1988, p.436) goes on to say that vaccination replaced the old practice of inoculation, which had been regularly used by the British and French in the slave trade since the mid-eighteenth century. Meanwhile, the Portuguese would have only experienced this method in Mozambique, against epidemic outbreaks, but not with the intent to “prevent the disease” before it appeared.

In terms of how the Angolans received the new technique, Manuel Barcia (2020, p.178) stresses that in the late 1840s Luandan authorities tried to maintain a quantity of vaccines sufficient to vaccinate all the Africans who were freed over a period of two years, which had been a common practice in Sierra Leone since the mid-1820s.

With this in mind, Sequeira Nobre’s shipment of vaccine material in 1810 seems novel, since the specialized historiography in this area states that the Jennerian vaccine was only applied in Angola in the 1820s. In this case, it is likely that the difficulties involved in transporting, preserving, and applying the vaccine initially were significant barriers to the success of the vaccine in places like Luanda and Benguela.

Nevertheless, Saldanha da Gama’s work to introduce the vaccine in Maranhão and later Angola, along with the influence of Bahian authorities (specifically the governor of Bahia and businessman Francisco Ignácio de Sequeira Nobre), highlights the connection between the Portuguese colonies and the circulation of health practices, often driven by figures with economic and political interests. In both cases, the use of enslaved people, either to propagate the vaccine material or as priority targets for vaccination, reveals the colonial dynamics of exploitation while also reflecting efforts to combat one of the greatest epidemic threats of the period.

The reports by agents of the Portuguese bureaucracy who worked in Bahia, Maranhão and Angola reveal the dynamics surrounding the vaccine’s movement along with details about transport and dissemination of the “vaccine humor” between the captaincies in Brazil and Africa. However, the involvement of men like Sequeira Nobre and Caldeira Brant during the early days of the vaccine should be analyzed based on their need to maintain their business ventures as well as royal incentives to implement it. Sequeira Nobre is emblematic because his gains were twofold: his slaves were vaccinated, and he also transported the vaccine in the glass slides he produced.

The networks of relationships established in the early nineteenth century to implement vaccination in the colonial territories involved merchants, governors, physicians and laypeople, as well as enslaved individuals whose bodies were used for the first experiments in immunization against smallpox in Brazil. While we cannot fully glimpse the extent of vaccination or the impact of this immunization on the disease’s control in Angola, we can state that the animal vaccine, which first arrived during the first decade of the nineteenth century via the arm-to-arm method or in glass slides, reached Angola from Brazil.

## Final considerations

The shipment of smallpox material to African territories, in human bodies or in glass slides, offers a range of possibilities for rethinking the history of the vaccine and the role of Bahia as a center for producing and propagating this resource. While the use of enslaved boys was part of the dominant logic of the time (since these individuals were embedded in networks of slaveholding relations), Bahia’s position as a center for disseminating the smallpox vaccine is novel. The ship Bom Despacho’s voyage to Lisbon did more than just ensure that enslaved people in Brazil’s captaincies were vaccinated: it also brought the vaccine to people in Africa, showing that the connections between these places during the colonial period extended beyond commercial trade.

Research in the documentary sources demonstrated the agency of slaveowners in the search for solutions to combat smallpox in the early nineteenth century. As seen in places like Havana, Cuba during this same period, enslaved subjects played a dual role in the early days of the vaccine in Brazil. Enslaved black boys were sent to Lisbon, where they were vaccinated and brought back to Salvador, where they were used to transport the immunization agent within the country.

The existing scientific production in colonial locations helped refine the science related to preventing epidemics, even in periods when this knowledge lost ground, such as the eighteenth and nineteenth centuries. We can therefore state that the ideas of Edward Jenner were accepted by Brazilian elites from the earliest days, not only because of their interest in protecting the cities from the many epidemic outbreaks of smallpox that occurred, but above all to secure their financial investments in enslaved people.

At the same time, the networks of relationships involving the vaccine in colonial territories such as Bahia and Angola demonstrate wider-ranging connections. The vaccine circulated in various territories through both the arm-to-arm method as well as glass slides. But the role of the Portuguese Crown in this process seems less intense, since the documentation indicates an active role played by slaveowners and local governors.

In any case, enslaved individuals undeniably played an essential role in bringing the vaccine to Brazil and subsequently Angola during the first decade of the nineteenth century, and their bodies were also utilized in this process: they were among the first people vaccinated in Portugal’s overseas territories and were implemented in networks to disseminate this prophylaxis within these territories.

Analysis of the measures taken to combat a lethal disease such as smallpox, which has been responsible for numerous epidemics throughout history, clearly reveals the importance of circulation of scientific knowledge in colonial lands, along with adaptation and development on the part of individuals in history to “prevent” the devastating effects of epidemic diseases. In this way, our examination of this topic addressed the history of science in the territories dominated by the Portuguese Empire while it also focused on specific details of slaveholding and hierarchical societies.

Broader knowledge of this scenario contributes to the understanding of the intersections involving health, science, and slavery in the fight against epidemics and the development of solutions to contain these diseases throughout history. Moreover, our analysis of the smallpox vaccine revealed a colony which took a more active position with regard to the conflicts of that time, rather than one which was passive and received ready-made ideas. But in the process of adapting the novelties surrounding experimental science, slavery and social relations of domination created the fundamental legacy for understanding aspects related to the arrival and subsequent circulation of the vaccine in spaces controlled by the Portuguese Empire in the early nineteenth century.

## Data Availability

Not deposited in a data repository.

## NOTES

1 Variolation was already known as the main tool in the fight against this disease in various regions during the seventeenth and eighteenth centuries (Sheridan, 1985). Eugenia Herbert (1975) stated that human pus was used for inoculation in various parts of Africa, and that use of this “remedy” in the Americas during the eighteenth and nineteenth centuries was the result of cultural exchanges involving the trafficking of enslaved people from the African continent.

2 Smallpox commonly killed a third of infected people; survivors were often left blind or scarred (Gazeta, 2006).

3 Dauril Alden and Joseph Miller (1987, p.195) presented data on the origins and dissemination of smallpox in Brazil, indicating transatlantic trafficking in slaves as a potential transmitter of diseases in the Americas. These authors claim that smallpox was a main cause of death during the ocean crossings: in other words, the black population was most significantly affected by the disease.

4 Importantly, indigenous peoples were also part of the group targeted by smallpox inoculation and vaccination practices during the early nineteenth century. Vaccination, which initially targeted enslaved people, also extended to other vulnerable populations, as noted by Barbosa (2019). In 1799, Prince Regent Dom João VI issued an order “to introduce and promote inoculation in black and Indian children” (Hochman, Sousa, 2022; Brizola, 2022, p.244).

5 Felisberto Caldeira Brant was informed of the results of the Spanish vaccine expedition in the Americas. The experiment involving the Brazilian boys, with support from the Portuguese royalty, shares similarities with the method adopted by the physicians Francisco Javier de Balmis and José Salvani, who left La Coruña in Spain in 1803 with 22 boys carrying smallpox material in their bodies (Ramírez Martín, 2002). The Royal Philanthropic Vaccine Expedition is considered the first and most significant attempt at mass vaccination in history, since it reached nearly all of South America (except Brazil) as well as locations in Africa and Asia (Balaguer I Periguell, Ballester, 2003; Ramírez Martín, 2002; Tuells, Duro Torrijos, 2015).

6 For details on the introduction of the smallpox vaccine in Portugal, see Barreiros (2014, p.218) and Portugal (2018, p.42).

7 According to Renato Tourinho Dantas (out. 1978), José Avelino Barbosa, who first applied the vaccination in Bahia, was one of a select group of Brazilian physicians who were trained abroad. He graduated in medicine from the University of Edinburgh, and began teaching classes at the Surgical Medical College in Bahia in 1815. He was also socially and politically active at the time of Brazil’s independence, and was elected Bahia’s provincial deputy in 1826 (Oliveira, 1992, p.34).

8 Maria Gabriela Marinho (2015, p.156, 157) described these “sanitary exchanges” as the information related to “health” that circulated between the captaincies and the royal court during the early nineteenth century, as well as the exchanges of the governors themselves and their respective experiences involving smallpox inoculation practices. Considering this wide-ranging circulation which can be seen during analysis of the documents in the various archives, this author identifies a specifically sanitary tendency in the colonial administration.
