# Supplementary material for: In arms and on slides: slavery-related interests in the dissemination of smallpox vaccine in Brazil and Angola, 1804-1810
Source: Hist Cienc Saude Manguinhos. 2026 Aug 7;33(Suppl 1):e2026027. doi: 10.1590/S0104-59702026000100027en (PMC13450909; doi:10.1590/S0104-59702026000100027en)
Supplement: Translation

# Translation: Entre braços e lâminas: interesses escravistas na distribuição da vacina antivariólica no Brasil e em Angola, 1804-1810

**DOI:** 10.1590/S0104-59702026000100027

**Authors:** Jaqueline Hasan Brizola, Gutiele Gonçalves dos Santos

**Affiliations:** i Pesquisadora de pós-doutorado, Programa de Pós-Graduação em História das Ciências e da Saúde/Casa de Oswaldo Cruz/Fiocruz. Rio de Janeiro – RJ – Brasil brizajaque@gmail.com; ii Doutoranda, Programa de Pós-Graduação em História das Ciências e da Saúde/Casa de Oswaldo Cruz/Fiocruz. Rio de Janeiro – RJ – Brasil gutielegoncalves12@gmail.com

**Keywords:** Vacina, Varíola, Escravidão, Bahia, Angola

## Abstract

Este artigo aborda interseções entre vacinação e escravidão no Brasil colonial, destacando a chegada da vacina antivariólica à Bahia e sua disseminação para outras regiões do Império português, incluindo localidades na África. A história de meninos escravizados, prioritariamente inoculados e vacinados entre o final do século XVIII e o início do século XIX, ganha destaque mediante a análise de fontes bibliográficas e documentais que ilustram a atuação de agentes históricos como Felisberto Caldeira Brant, Francisco da Cunha Menezes e Francisco Ignácio de Sequeira Nobre. A atuação desses personagens revela a iniciativa de senhores escravistas na busca por solução contra a varíola, evidenciando complexas relações entre escravidão, ciência e saúde.

Em 1805, poucos anos após o anúncio da vacina antivariólica na Europa, os debates em torno do transporte e execução do método preventivo estavam presentes em localidades do Brasil e da África. Este artigo explora as interseções entre vacinação e escravidão na primeira década do século XIX, enfocando os processos envolvendo a chegada da vacina à Bahia em 1804 e posterior envio do material varioloso para outras partes do Império português, incluindo Angola. Além de problematizar aspectos relacionados à recepção da vacina – que, no Brasil, só foi possível mediante a utilização de braços de sujeitos escravizados e indígenas –, buscaremos demonstrar as ações estabelecidas entre os comerciantes que atuavam na Bahia e burocratas a serviço do Reino de Portugal no Brasil e em Angola para a distribuição da vacinação em territórios coloniais.

A técnica da vacinação, proposta por Edward Jenner, baseava-se na observação de que pessoas que haviam entrado em contato com o vírus bovino (*cowpox*) não contraíam a varíola humana (*smallpox*). Ao inocular um menino de 8 anos com o “pus bovino” e expô-lo posteriormente à varíola, o médico demonstrou a eficácia da vacina, que recebeu esse nome devido à sua origem nas pústulas da vaca (Chalhoub, 1996, p.136). Os experimentos de Jenner rapidamente se tornaram conhecidos em diversas partes do mundo, e a antiga prática da variolização – que envolvia a inoculação do vírus da varíola humana – foi gradualmente substituída pela vacinação, baseada na introdução do vírus da varíola bovina (Esparza, 2020).^1^

No continente americano, a varíola causou estragos desde os primeiros contatos com os europeus no século XV, sendo considerada uma arma mais letal que a pólvora para o extermínio dos povos originários (Crosby, 2001; Malvido, 2003; Anzolin, 2014).^2^ Contudo, no caso do Brasil, a responsabilização pela introdução de novas epidemias foi comumente atribuída aos escravizados que chegavam da África (Carvalho, 2006). Devido à situação de endemia da doença no continente africano, sobretudo ao longo do século XVIII, criou-se a ideia de que esses sujeitos eram os grandes responsáveis pelo quadro de descontrole da varíola no Brasil (Alden, Miller, 1987).^3^

Diferentemente do que ocorreu em outros países, onde integrantes da nobreza levaram seus filhos para receberem a vacina (Duro Torrijos, 2014, p.320), o cenário no Brasil aponta para a execução dos primeiros testes em escravizados e indígenas.^4^ Com base em documentos oficiais presentes na Biblioteca Nacional do Rio de Janeiro, sabe-se que Felisberto Caldeira Brant, comerciante mineiro que atuava na Bahia, enviou sete meninos de sua propriedade a Lisboa em 1804 para que fossem inoculados com o *cowpox* (Chalhoub, 1996; Portugal, 2018; Brizola, 2022). De volta ao Brasil, os meninos teriam viabilizado o primeiro plano de vacinação no país.^5^

Além de Caldeira Brant, o comerciante de vidros Francisco Ignácio de Sequeira Nobre e os governadores da Bahia e do Maranhão, Francisco da Cunha Menezes e Antônio de Saldanha da Gama, aparecem como importantes interlocutores para a chegada da vacina no Brasil e posterior envio para Angola. Analisando a trajetória desses agentes, busca-se demonstrar os conflitos e interesses envolvendo a imunização, a movimentação e a elaboração de sujeitos em torno das “descobertas científicas” da época, as formas socialmente aceitas de prevenir a varíola, além de descortinar a importância da escravidão e das relações escravistas para a história das ciências em territórios coloniais administrados pelo império português em princípios do século XIX.

O recorte temporal selecionado para a presente análise compreende um período de transição quanto à interpretação das doenças ou as formas de combatê-las, pois, na segunda metade do século XVIII, a influência do iluminismo conferiu à medicina e aos médicos diplomados lugar de destaque na “prevenção”, diagnóstico e “cura” das doenças (Walker, 2013). Quanto à documentação utilizada, devemos referenciar a correspondência oficial trocada entre Francisco da Cunha Menezes e João Rodrigues de Sá e Melo, o visconde de Anadia, em Lisboa, que nos auxilia na compreensão de aspectos relacionados à introdução da vacina no Brasil a partir de 1804. Além disso, outros documentos presentes no Arquivo Público do Estado da Bahia e no Arquivo Histórico Ultramarino em Lisboa contribuem para o entendimento dos processos relacionados ao transporte e envio do *cowpox* para outras localidades, seja por meio da técnica conhecida como “braço a braço” ou de lâminas de vidro confeccionadas para melhor acondicionar o pus vacínico (Fernandes, 2003).

As informações extraídas dos diferentes documentos de que dispomos compuseram uma série de dados reunidos e analisados a partir de metodologia qualitativa, que conferiu significado aos discursos dos agentes envolvidos com a vacinação antivariólica sem, no entanto, desconsiderar que tais discursos carregavam determinadas intencionalidades. Paralelo à análise das fontes, tornou-se necessário enxergar os processos envolvendo a vacina antivariólica a partir da lupa da história social em diálogo com aportes teóricos pós-coloniais. Conforme a definição de Bhabha (1998, p.239), a perspectiva pós-colonial tem suas raízes no testemunho dos países tidos como periféricos, onde se exaltam as contribuições das “minorias” dentro das redes de relações que se formam entre o que se convencionou chamar de centro e periferia.

As referências à Bahia como centro de distribuição da vacina nos levam a reconsiderar o papel dos agentes que atuavam nessa localidade, que não estão apenas subordinados às decisões do Reino, mas atuando em favor de seus negócios e da empresa escravista. Embora comerciantes como Felisberto Caldeira Brant e Francisco Ignácio de Sequeira Nobre estivessem alinhados com as ideias vindas de Portugal, suas ações evidenciam a estreita relação entre os interesses escravistas e o projeto de vacinação no Brasil e em Angola já nos primeiros anos do século XIX.

Tendo em conta esse cenário, algumas questões são problematizadas no presente artigo. Como a experiência da escravidão e os interesses escravistas influenciaram o desenvolvimento das práticas adotadas para “prevenir” a varíola em territórios coloniais como Bahia e Angola? Quais acordos foram estabelecidos para a chegada da vacina jenneriana ao Brasil na primeira década do século XIX, e quem foram os agentes envolvidos com a difusão da vacina? E, ainda, de que maneira os corpos de escravizados foram utilizados para a propagação da vacina em territórios controlados pelo Império português na América e na África?

A história da vacina antivariólica mobiliza múltiplas variáveis no âmbito científico, político ou social; todavia, ainda há lacunas no que se refere ao entendimento dos impactos gerados no combate à enfermidade em contextos escravistas. Nesse caso, as práticas em torno dessa vacina na Bahia ou em Angola oferecem um terreno fértil para o entendimento das conexões entre saúde, medicina e escravidão. As questões temporais e geográficas aqui delimitadas nos permitem compreender a dinâmica de adoção, incentivo e envio da vacina jenneriana por meio do Atlântico em um período notabilizado pela difusão de práticas científicas no combate às enfermidades contagiosas.

Os estudos envolvendo epidemias, ciência e escravidão avançaram ao longo das últimas décadas, aportando contribuições ao conhecimento histórico e oferecendo explicações sobre os métodos utilizados para combater quadros epidêmicos que frequentemente assolavam as populações do continente americano desde os primeiros contatos entre os europeus e os povos originários da África e das Américas (Sheridan, 1985; Alden, Miller, 1987). Os debates que emergem nessas áreas, sobretudo a partir da década de 1980, incorporam as múltiplas experiências históricas reconhecendo saúde e doença dentro de uma construção e um enquadramento social que leva em consideração o tempo histórico, os grupos sociais e os indivíduos envolvidos (Rosenberg, 1992).

Ao longo das últimas décadas, autores como Derrick Baxby (1996), Donald Hopkins (2002) e Pierre Darmon (1986) têm destacado que práticas semelhantes à vacinação contra a varíola já eram utilizadas por populações rurais em diferentes localidades, mesmo antes da oficialização da vacina de Jenner como método preventivo, revelando como essas iniciativas foram apropriadas por projetos imperiais entre os séculos XVIII e XIX.

Rechaçando a ideia de triunfalismo europeu, Michael Bennett (2020, p.6) afirma que a difusão da vacinação antivariólica ao redor do globo foi um esforço humanitário. Ao questionar os pressupostos do atraso e da dependência colonial, o autor chama a atenção para o papel da escravidão na disseminação da vacina no continente americano, já que, em princípios do século XIX, a maioria das localidades por onde a vacina passou no ultramar eram entrepostos escravistas (Bennett, 2020, p.319). A obra de Bennett merece destaque por realizar uma análise global sobre a realidade da vacina em diversos países, incluindo o Brasil.

Na África, onde a varíola era uma doença permanente, aprender esse novo método preventivo significava que muitas vidas daqueles que eram enviados para o comércio transatlântico de escravizados poderiam ser salvas. O denso estudo sobre Benguela, realizado pela historiadora Mariana Cândido (2013), demonstra que os acontecimentos no Brasil afetavam a costa africana em termos políticos e sociais, já que o comércio de escravizados aproximou o Brasil e a África para além das trocas comerciais. A autora, que sublinhou a importância do Atlântico Sul como espaço de circulação de pessoas, ideias e culturas, não deixou de reconhecer a gravidade das epidemias de varíola, que se manifestaram de forma recorrente em Benguela ao longo do século XIX.

Por essa razão, segundo Jill Dias (1981, p.364), os membros mais poderosos da comunidade colonial agiam rapidamente para evitar a morte de cativos. Conforme destacou a autora, já na década de 1860, o empresário João Caetano Sarmento, atuante em Angola, teria mandado vacinar com o *cowpox* todos aqueles que se encontrassem sob sua propriedade em Luanda, Benguela Velha e Dombe Grande no início de uma epidemia de varíola (Dias, 1981, p.364). Nesse caso, a vacinação de escravizados africanos, inicialmente apoiada por negociantes da capitania da Bahia, continuou a ser adotada também pelos proprietários de escravos locais em diferentes regiões de Angola.

Joseph Miller (1988, p.213) afirma que as aplicações efetivas de novas tecnologias médicas reconhecidas como eficazes contra as doenças tropicais foram paulatinamente incorporadas à cultura de muitos africanos que viviam na costa ocidental, sobretudo em Angola, cujos principais portos, Luanda e Benguela, mantinham contato direto com viajantes oriundos da América e da Europa. O autor sustenta, no entanto, que a vacina jenneriana teria chegado à Angola apenas na década de 1820, tendo reduzido significativamente a variação anual da mortalidade entre os escravizados (Miller, 1988, p.436).

No Brasil, após os estudos pioneiros de Sidney Chalhoub (1996) e Tânia Fernandes (2003) que analisaram aspectos sociais e institucionais das práticas de variolização e vacinação, uma nova geração de historiadores e historiadoras vêm problematizando a temática da vacina antivariólica e seu impacto para o controle da doença (Pimenta, Gomes, Kodama, 2018; Portugal, 2018; Brizola, 2022; Santos, 10 maio 2024). Fillipe Portugal (2018), que estudou a introdução da vacinação no Império português a partir dos casos do Brasil e Portugal, destacou o pioneirismo da Bahia, do Rio de Janeiro e de São Paulo para a execução da vacina com o *cowpox* a partir de 1803. O papel desempenhado por comerciantes escravistas da Bahia nos primórdios da vacinação no Brasil não foi ignorado pelo autor, que analisou também a atuação de médicos vacinadores como José Avelino Barbosa, em Salvador, e Hércules Otaviano Muzzi, no Rio de Janeiro.

Sobre a chegada da vacina na província da Bahia e na cidade de Salvador, Hochman e Souza (2022) argumentaram que esses locais foram uma espécie de centros de inovação em termos de resposta pública à varíola. Ao analisar as percepções e ações do poder público no processo de produção e conservação do pus variólico, os autores oferecem uma importante contribuição ao entendimento do papel desempenhado pela Bahia, uma vez que a capitania passa a ser entendida enquanto lócus de distribuição e administração de vacinas na primeira década do século XIX. Hochman e Souza (2022, p.3432) mencionam, ainda, que da Bahia teriam saído remessas de vacina para Angola, segundo notícia veiculada pela *Gazeta do Rio de Janeiro* em 1811.

O pioneirismo da Bahia como centro de distribuição da vacina nos primeiros tempos também foi destacado pela historiadora Maria Gabriela Marinho (2015, p.155). A autora analisou o circuito de comunicação relacionado à vacinação antivariólica entre a Corte e os governadores de territórios ultramarinos. Investigando a documentação presente no Arquivo Histórico Ultramarino, Marinho concluiu que, entre fins do século XVIII e princípios do século XIX, existia uma espécie de administração sanitária colonial capaz de orientar e coordenar, primeiramente, os processos de inoculação do pus variólico e, posteriormente, a vacinação com o *cowpox.* Marinho sugere que a Bahia pode ter assumido alguma relevância como “entreposto” de redistribuição da vacina e das instruções emanadas da Corte.

Concordando com Marinho, Hochman e Souza quanto ao pioneirismo da Bahia nos processos de distribuição da vacina entre capitanias do Brasil, destacamos as interlocuções entre o Brasil e a África no processo de difusão da vacina antivariólica pelo método “braço a braço” ou pelo transporte do pus variólico em lâminas de vidro.

Como veremos a partir de agora, as redes de relações que proporcionaram a difusão da imunização nos primeiros tempos envolveram comerciantes, médicos e burocratas do Reino. A análise da atuação desses sujeitos é representativa dos combates travados contra uma doença que causou enormes estragos ao longo da história, mas também demonstra os interesses escravistas, mediando as ações em termos de “prevenção” à varíola. A adaptação ao contexto colonial engendrou formas particulares de aplicar a ciência, dado que os primeiros experimentos com a vacina no Brasil só foram possíveis mediante a utilização de corpos de indígenas e escravizados.

## Circulação de conhecimento e interesses escravistas na difusão e no transporte da vacina

A chegada da vacina no Brasil ocorre concomitantemente a sua introdução em Portugal.^6^ Seguindo uma tendência verificada em outros reinos e buscando legitimar a nova técnica, o príncipe regente dom João VI mandou vacinar seus filhos em 1804, e outros nobres lusitanos seguiram o caminho do monarca. Desse modo, a vacina animal rapidamente substituiu a inoculação da varíola humana, e a casa dos Bragança passou a executar uma “política absolutista em favor da vacinação” (Portugal, 2018, p.42).

Na Bahia, a vacina desembarcou em 1804, transportada por meio dos corpos de sete meninos escravizados. De acordo com Camargo (2008) e Brizola (2022), essa não teria sido a primeira vez que a vacina de Jenner foi utilizada no Brasil, pois há registros oficiais de vacinação ocorrendo em São Paulo desde 1803. Esses documentos, preservados tanto no Arquivo Histórico Municipal de São Paulo quanto no Arquivo Ultramarino de Lisboa, indicam uma circulação inicial do imunizante entre os paulistas antes da chegada formal à Bahia.

Entretanto, é inegável que, a partir da iniciativa ocorrida na Bahia, a vacinação ganhou maior destaque no Brasil. Francisco da Cunha Menezes, governador da Bahia em 1805, oferece detalhes da chegada da vacina, demonstrando a utilização de braços escravizados no processo:

Tendo chegado a este porto no dia 30 de dezembro passado, o navio Bom Despacho, que transportava os sete negrinhos d’aqui enviados para conduzirem a vacina, por alguns negociantes d’esta cidade, no dia subsequente se deu logo princípio ao uso deste preservativo das bexigas fazendo na minha presença administrar a vacinação com o humor do último dos sete vacinados a mais de cem pessoas de diversas idades pelo cirurgião daquele navio, Manoel Moreira da Rosa e o médico José Avelino Barbosa (Ofício..., 4 jan. 1805).

Pelo método de “braço a braço”, que consistia em vacinar o indivíduo com fluido fresco da pústula de outra pessoa no pico da reação, a vacina chegou à Bahia em 1804, conforme relato do governador daquela capitania. Nota-se que, com a utilização de corpos de meninos escravizados para o transporte do pus vacínico, foi possível vacinar outras pessoas naquele momento, já que a expedição acompanhada pelo médico José Avelino Barbosa e pelo cirurgião Manoel Moreira Rosa, ao que parece, obteve sucesso em seus propósitos iniciais.

A pesquisa em documentos manuscritos revelou as redes de relações estabelecidas entre agentes do Império português que atuavam nas capitanias do Brasil e grupos de particulares interessados em praticar a vacinação. Do ponto de vista da história social, esse cenário contribui para o entendimento dos interesses escravistas e do critério da raça mediando as diferentes relações que se estabelecem durante o período colonial, incluindo as descobertas científicas mais aclamadas naquele momento.

Nesse caso, a partir de 1804, os processos envolvendo a vacina obedecem a diferentes interesses que, para além de combater o mal das bexigas, dialogavam com os propósitos escravistas, pois os homens que se dedicaram à propagação da vacina nos primeiros tempos eram ricos comerciantes e proprietários de escravizados. Essa tendência foi observada em outros espaços estratégicos no continente americano. O historiador cubano Beldarraín Chaple (2015) observou que, a partir de 1804, havia maior preocupação com a vacinação dos escravizados em Cuba, sobretudo daqueles recém-chegados da África, já que a varíola representava um prejuízo econômico incalculável para os comerciantes e senhores escravistas na ilha caribenha.

Em relação à chegada da vacina à Bahia, Michael Bennett (2020, p.314) destaca a centralidade da figura do médico José Avelino Barbosa, que esteve na Inglaterra em 1803 e obteve amostras da vacina. A interlocução entre Barbosa e Felisberto Caldeira Brant, segundo o autor, levou à organização da expedição dos meninos escravizados a Lisboa em 1804 com o apoio do governador da Bahia, Francisco da Cunha Menezes. Embora não possamos desconsiderar a atuação de Menezes para a chegada e o transporte do *cowpox* no Brasil, a documentação investigada aponta para o protagonismo de outros agentes.

Nesse caso, a atuação de indivíduos diretamente ligados ao comércio de africanos para que a vacinação ocorresse no Brasil durante os primeiros anos do século XIX foi bastante importante, o que inviabiliza a ideia de uma colônia passiva, que recebia as ordens de forma vertical sem qualquer tipo de negociação e mediação com a metrópole. Os agentes envolvidos com a vacina no Brasil tiveram relativa autonomia para aplicar as medidas necessárias para o funcionamento da imunização nos primeiros tempos.

Ressalta-se que a ideia de passividade do Brasil em relação à metrópole no antigo sistema colonial é tema contestado pela historiografia brasileira há mais de duas décadas, sendo um dos principais marcos dessa discussão a publicação do livro *Antigo regime nos trópicos* (Fragoso, Bicalho, Gouvêa, 2001). Quando observamos a autonomia dos agentes envolvidos com a vacina no Brasil, fornecemos um exemplo daquilo que Antônio Manuel Hespanha (1994) chamou de pluralidade dos laços políticos nas relações entre o poder local e a metrópole, em que as ações voltadas aos interesses dos poderosos locais não eram incomuns, independentemente do consentimento da Coroa.

Em carta, remetida por Francisco da Cunha Menezes ao Reino, o governador da Bahia informa sobre a atuação do médico José Avelino Barbosa, responsável pela vacinação naquela capitania. Após as primeiras aplicações da vacina na Bahia, Barbosa teria enviado suas observações ao próprio Edward Jenner. Dirigindo-se ao príncipe regente em 1805, Menezes informa que

o médico José Avelino Barbosa, a quem sua alteza real foi servido [*sic*] encarregar a direção da vacinação nesta capitania me apresenta essas duas cartas uma para o cirurgião mor da armada Teodoro Ferreira de Aguiar, e outra para o Doutor Jenner, em Londres, em que lhe participa algumas observações suas a respeito da vacina neste país (Ofício..., 26 jan. 1805).

Para além da utilização de meninos escravizados na inoculação e no transporte do material varioloso entre Bahia, Rio de Janeiro e Angola, médicos encarregados de aplicar a vacina, como José Avelino Barbosa, estavam divulgando suas experiências em correspondências enviadas para fora do Brasil. Segundo as informações relatadas ao reino pelo governador da Bahia em 1805, Edward Jenner teria recebido uma carta de José Avelino que continha dados das primeiras experiências com o *cowpox* no Brasil.^7^

Não conhecemos o local de guarda da referida correspondência nem seu conteúdo, mas, de posse das informações fornecidas por Menezes, não seria exagero afirmar que as atividades realizadas no Brasil e, especialmente, na Bahia para a aplicação da vacina mereceram destaque naquele momento. De outro modo, não seria possível uma interlocução com Jenner em Londres.

De qualquer maneira, e conforme o relato do próprio governador Francisco da Cunha Menezes, em 1805, 1.300 pessoas haviam sido vacinadas na Bahia, incluindo lugares do Recôncavo baiano, precisamente nos engenhos de açúcar (Ofício..., 1 jun. 1805). Essa última informação nos leva a crer que os cativos das fazendas açucareiras do nordeste do Brasil estavam entre os grupos prioritários para os primeiros testes com a vacina na capitania da Bahia, já que nesses espaços havia dezenas, por vezes centenas de escravizados que trabalhavam na produção de açúcar exportado pelo Brasil.

Quanto ao transporte do pus variólico, devemos destacar a atuação de Francisco Ignácio de Sequeira Nobre, rico comerciante escravista que empreendeu no setor de fabricação de vidros em princípios do século XIX. Sua atuação no transporte do pus vacínico está intimamente ligada aos seus negócios, já que solicitou vantagens em Lisboa com o objetivo de expandir seu comércio.

Na obra *Observações sobre a franqueza da indústria e estabelecimento de fábricas no Brasil*, publicada em 1810, José da Silva Lisboa (1999, p.75) menciona que, segundo o alvará de 28 de abril de 1809, aqueles que fossem “inventores e introdutores de alguma nova máquina ou invenção nas artes” teriam o privilégio de exclusividade no negócio por quatorze anos, garantindo ao proprietário um “mercado extenso, progressivo e lucroso”.

Esses termos caracterizaram exatamente o comércio de Francisco Ignácio de Sequeira Nobre, pois, além de atuar no comércio de escravizados para a Bahia no início do século XIX, fundou, naquela capitania, em 1810, a primeira Fábrica de Vidros do Brasil, após receber autorização da Coroa. A fábrica produzia uma ampla variedade de itens, desde objetos decorativos até utensílios médicos, como frascos de remédios e ventosas (Notícia..., 1813).

Ao recuperar a história do transporte do pus vacínico quando o material chegou ao Brasil, observamos a atuação de Sequeira Nobre na disseminação da vacinação contra a varíola, já que é mencionado em documentos oficiais como responsável por transportar material vacínico, inclusive para Angola, com o intuito de vacinar pessoas escravizadas ainda em território africano. Esse procedimento visava minimizar os riscos de surtos de varíola, tanto durante a viagem nos navios negreiros quanto ao chegarem aos portos de Salvador, onde as epidemias poderiam se espalhar rapidamente entre os recém-chegados e a população local.

Devido à dificuldade de transportar pessoas inoculadas com o vírus e aos riscos associados a essa prática, o transporte do material vacínico em lâminas de vidro foi amplamente adotado no Brasil, especialmente após a formação da Junta Vacínica da Corte em 1811 (Fernandes, 2010). Esse método, conhecido popularmente como “matéria seca”, consistia em colocar o pus retirado do braço do doente entre duas placas de vidro, onde secava rapidamente. A técnica facilitava o transporte e o envio do material para outras regiões. Para realizar a vacinação com a matéria seca, era necessário diluir o material em uma pequena quantidade de água fria, agitando-o com a ponta da lanceta por alguns minutos até que a mistura adquirisse uma leve opacidade (Chernoviz, 1851, p.455).

Por prestar o serviço de fabricar vidros que poderiam ser utilizados para transportar remédios, incluindo a matéria seca da vacina, Sequeira Nobre solicitou ao rei o direito de exclusividade na produção e comercialização de vidros por um período de vinte anos no Brasil. Em resposta, o monarca não concedeu o privilégio exatamente como solicitado, mas ofereceu algumas garantias em reconhecimento aos benefícios que seus negócios traziam para o Reino (Carta do príncipe..., 1 set. 1810).

A atuação de sujeitos como Sequeira Nobre e Felisberto Caldeira Brant no empreendimento da vacina expõe as vicissitudes das relações sociais de trocas ou concessões de privilégios e mercês, típicas das sociedades do Antigo Regime. Para promover a vacina nos domínios ultramarinos, o Reino contou com a ajuda de particulares que, prestando serviços ao bem comum, garantiam seus privilégios. Com efeito, é preciso sublinhar a colaboração de Lisboa com o transporte e execução da vacina, tanto no Brasil quanto em outras localidades do Império (Saavedra, 2004).

Nota-se, entretanto, que o interesse de Sequeira Nobre na difusão da vacina apresentava também motivações econômicas. Vacinar os escravizados nos portos de origem antes da travessia atlântica minimizava os riscos de surtos a bordo, evitando grandes prejuízos financeiros para os comerciantes escravistas. Além disso, uma vez que os escravizados desembarcavam e eram levados aos armazéns de venda, garantir que estivessem protegidos contra a varíola era essencial para o comércio. Evitava a disseminação de epidemias e preservava a “saúde pública” e os lucros dos comerciantes envolvidos no tráfico atlântico. Desse modo, a “saúde” dos escravizados era instrumentalizada em função de interesses comerciais, visto que as práticas de vacinação estiveram vinculadas ao sistema escravista e às dinâmicas econômicas da época.

## Uma remessa para Angola: dinâmicas escravistas no combate à varíola

Como vimos, a importância do comerciante de vidros Francisco Ignácio de Sequeira Nobre para o transporte do material vacínico superou as capitanias de Rio de Janeiro, São Paulo ou Minas Gerais, onde matinha postos de abastecimento importantes para seus negócios. Com o domínio da técnica de vacinação com o *cowpox*, o governador da Bahia teria enviado o material varioloso em lâminas de vidro para outras capitanias do Brasil e para Angola. Segundo Menezes,

em ofício de 15 de março desse ano, tinha eu participado a vossa excelência não só haver mandado humor vacínico em vidros aos governadores e capitães gerais do Reino de Angola e São Paulo como também haver mandado no [*sic*] de sua majestade que transportava o tabaco da Índia para o Rio de Janeiro a Jorge Francisco Machado, ajudante de cirurgia do primeiro regimento de linha desta cidade com quatro rapazes para os ir vacinando durante a viagem (Carta de Francisco..., 1 jun. 1805).

As lâminas de vidro fabricadas por Sequeira Nobre eram uma opção no envio de material vacínico, inclusive para longas distâncias. O transporte da vacina por meio de braços de meninos escravizados, no entanto, ocorria paralelamente, garantindo a circulação do *cowpox* e de seus efeitos em localidades no Brasil e na África já nos primeiros anos do século XIX.

Sobre as epidemias na costa ocidental africana, Alden e Miller (1987, p.203) apresentam dados que comprovam a ocorrência da varíola em formato endêmico e epidêmico ao longo de todo o século XVII. Por essa razão, eram comuns as queixas de proprietários de escravizados, que relatavam ter perdido toda a sua força de trabalho em decorrência da doença. Em resposta a essa situação, as autoridades da Bahia chegaram a ordenar uma quarentena específica para os escravizados que chegavam de Angola, embora não tenham conseguido impor efetivamente essa restrição (Hochman, Souza, 2022, p.3431). Assim, dada a dificuldade de fiscalização e controle das embarcações, investir na vacinação dos africanos tornou-se a alternativa mais viável.

Outro agente importante que aparece no centro dos debates em relação à vacina no período investigado é Antônio de Saldanha da Gama, governador do Maranhão em 1805. Homem de confiança do Reino, posteriormente foi transferido para atuar em Angola, onde favoreceu as “trocas sanitárias” entre territórios coloniais.^8^ A prática de transferir governadores para diferentes localidades era recorrente no extenso Império português. Conforme argumentaram Gouvêa, Frazão e Santos (2004, p.103), algumas singularidades e alguns mecanismos específicos guiavam as práticas sociais, políticas e econômicas na sociedade portuguesa do Antigo Regime. Para os autores, a natureza multicontinental do Império português esteve entre as razões para que muitos altos oficiais prestassem serviços em diferentes territórios ultramarinos ao longo de suas carreiras.

Esse foi o caso de Saldanha da Gama, que atuou na distribuição da vacina animal em Angola após ter tomado conhecimento dos benefícios do imunizante. Antes de desembarcar em território africano, Saldanha da Gama obteve experiência com a vacina quando permaneceu no posto de governador do Maranhão. Escrevendo de São Luís, o governador relata, em 1805, a experiência que teve com a vacina enviada da Inglaterra à capital do Maranhão. Referindo-se à atuação do governador da Bahia a favor da vacina, Saldanha da Gama afirma que

instigado pelo seu conhecido zelo e patriotismo enviou um bergantim [ao Maranhão] e fez inocular alguns escravos na presença do mestre do dito bergantim ensinando e mostrando os instrumentos para se continuar a dita operação acompanhando esta remessa com matéria seca conservada entre vidros (Ofício…, 24 jun. 1805).

Ainda segundo o relato de Saldanha da Gama, quando a matéria seca enviada pelo governador da Bahia chegou ao Maranhão, foi no mesmo dia aplicada nas “pessoas escravas que ainda não tiveram bexigas”. Contudo, os “desejos do incansável governador foram malogrados”, porque “o mestre da embarcação ou por injúria ou por outras circunstâncias alega ter deixado secar a matéria ao passar para os corpos” (Ofício..., 24 jun. 1805).

Essa havia sido a segunda experiência de vacinação mal lograda no governo de Antônio de Saldanha da Gama. A primeira tentativa foi a compra de “seis moleques da cidade da Bahia, para que fossem de Salvador a São Luís, por vias terrestres, acompanhados de um hábil cirurgião que deveria providenciar que chegassem à cidade com o pus da vacina em estado de propagar na capitania” (Informação..., 27 jan. 1806).

Como se vê, tanto pelo método de “braço a braço” como por meio de lâminas de vidro, a utilização de escravizados era parte integrante dos planos de vacinação antivariólica, não apenas na Bahia. Ao anunciar a compra de “seis moleques” para que a vacina chegasse ao Maranhão, Saldanha da Gama demonstra que utilizar meninos escravizados pode ter se tornado uma prática recorrente para o transporte interno da vacina em princípios do século XIX.

Em 1806, Saldanha da Gama deixou o governo do Maranhão para assumir o cargo de governador geral de Angola. Mesmo nessa transição, manteve sua atuação nas discussões sobre a vacinação. Já em Angola, em 1810, reforça, em uma de suas cartas, o incansável zelo de Francisco Ignácio de Sequeira Nobre, que enviou

um bergantim de guerra a fim de comunicar aquele país o pus vacínico para evitar as funestas consequências que no ano antecedente foram ceifadas [*sic*] pelo mal das bexigas. Sendo esta expedição promovida pelo dito Francisco Ignácio de Sequeira Nobre como me comunicou o governador naquela dita capitania e para o qual ele concorreu não só com diligências, mas até com despesas, motivando-me isso a dirigir-lhe os meus agradecimentos (Gama, 1 set. 1810).

A carta de Saldanha da Gama, entretanto, não fornece detalhes acerca do transporte do pus vacínico. Nesse caso, não é possível antever se em 1810 a vacina teria chegado a Angola por meio de meninos escravizados, repetindo a experiência da Bahia em 1804, ou se teria sido transportada em lâminas de vidro. O que sabemos, diante do relato do governador, é que o remédio chegou a Angola em princípios do século XIX, contando com a colaboração de Sequeira Nobre, proprietário da primeira fábrica de vidros do Brasil, que, ademais, mantinha negócios com o lucrativo comércio de escravizados entre as costas da África e do Brasil.

Há poucos relatos sobre a difusão da vacina jenneriana em Angola na primeira metade do século XIX. Joseph Miller afirma, no entanto, que a técnica de Jenner teria causado impactos importantes na diminuição da mortalidade daqueles sujeitos enviados compulsoriamente para América a partir de 1820. Ainda segundo Miller (1988, p.436), a vacinação substituiu a antiga prática da inoculação, que foi usada de forma recorrente por britânicos e franceses no comércio negreiro desde meados do século XVIII. Os portugueses, por sua vez, teriam experimentado o método apenas em Moçambique, diante de surtos epidêmicos, mas não com a intenção de “prevenir a doença” antes de seu aparecimento.

Quanto à recepção dos angolanos à nova técnica, Manuel Barcia (2020, p.178) sublinhou que, no final da década de 1840, as autoridades de Luanda tentaram manter vacinas suficientes para vacinar todos os africanos libertados num período de dois anos, algo que tinha sido uma prática comum em Serra Leoa desde meados da década de 1820.

Diante do exposto, o envio do material varioloso por Sequeira Nobre em 1810 aparece como novidade, pois a historiografia especializada no tema afirma que a vacina de Jenner só passou a ser ministrada em Angola na década de 1820. Nesse caso, é provável que, no primeiro momento, as dificuldades inerentes a transporte, conservação e aplicação da vacina tenham constituído barreiras importantes para o sucesso da vacina em localidades como Luanda e Benguela.

De qualquer maneira, a atuação de Saldanha da Gama na introdução da vacina no Maranhão e, posteriormente, em Angola, aliada à influência das autoridades baianas, especificamente a do governador da Bahia e do negociante Francisco Ignácio de Sequeira Nobre, evidencia a conexão entre as colônias portuguesas e a circulação de práticas de saúde, muitas vezes impulsionadas por figuras com interesses econômicos e políticos. Em ambos os casos, o uso de escravizados, seja como propagadores da vacina ou como alvo prioritário dela, revela as dinâmicas coloniais de exploração, ao mesmo tempo que refletem os esforços de combate a uma das maiores ameaças epidêmicas do período.

A partir do relato dos agentes da burocracia portuguesa atuando na Bahia, no Maranhão e em Angola, podemos entender as dinâmicas em torno da movimentação da vacina, assim como os detalhes sobre o transporte e a difusão do “humor vacínico” entre capitanias no Brasil e na África. No entanto, o envolvimento de homens como Sequeira Nobre e Caldeira Brant nos primeiros tempos da vacina deve ser analisado a partir da necessidade de ambos em manter seus negócios e do incentivo da Coroa para aplicar a medida. O caso de Sequeira Nobre é emblemático porque o comerciante ganhou duplamente: com a vacinação de seus escravizados e com o transporte da vacina nas lâminas de vidro que produzia.

As redes de relações que se estabeleceram em princípios do século XIX para a aplicação da vacina em territórios coloniais envolveram comerciantes, governadores, médicos e leigos, além de pessoas escravizadas que tiveram seus corpos utilizados para os primeiros experimentos de imunização contra a varíola no Brasil. No caso de Angola, não é possível vislumbrar a extensão da vacinação ou o impacto do imunizante no controle da doença em território africano. O que, de fato, se pode afirmar é que a vacina animal, transportada de braço a braço ou em lâminas de vidro, chegou a Angola na primeira década do século XIX, tendo como ponto de partida o Brasil.

## Considerações finais

O envio de material varioloso nos corpos de pessoas ou em lâminas de vidros para territórios na África abre um leque de possibilidades para repensarmos a história da vacina e do protagonismo da Bahia como centro gerador e propagador do remédio. Se, por um lado, a utilização dos meninos escravizados respeitava a lógica dominante na época, já que esses sujeitos estavam imersos em redes de relações escravistas, por outro, o protagonismo da Bahia como centro de propagação da vacina antivariólica aparece como uma novidade. A viagem do navio Bom Despacho a Lisboa não garantiu apenas a vacinação de sujeitos escravizados nas capitanias do Brasil. A partir desse empreendimento, pessoas na África receberam a vacina, o que demonstra que as conexões existentes entre essas localidades no período colonial extrapolavam as trocas comerciais.

A pesquisa em fontes documentais demonstrou a agência de senhores escravistas na busca por soluções de combate à varíola em princípios do século XIX. De modo semelhante ao que ocorreu em lugares como Havana, em Cuba, no mesmo período, os sujeitos escravizados cumpriram um duplo papel nos primórdios da vacina no Brasil. Meninos negros escravizados foram enviados para Lisboa, onde receberam a vacina, em seguida retornaram a Salvador e foram utilizados para o transporte interno do imunizante no país.

As produções científicas existentes em localidades coloniais contribuíram para o aprimoramento da ciência relacionada à prevenção de epidemias, inclusive em períodos recuados, como nos séculos XVIII e XIX. Com isso, afirma-se que as ideias de Edward Jenner foram aceitas por parte da elite no Brasil nos primeiros tempos, não apenas pelo interesse em preservar as cidades dos muitos surtos epidêmicos de varíola que ocorriam, mas, sobretudo, para assegurar o investimento financeiro em escravizados.

Por outro lado, as redes de relações envolvendo a vacina em territórios coloniais como Bahia e Angola demonstram conexões mais amplas. Seja pelo método de braço a braço ou armazenado em lâminas de vidro, o fluido vacinal circulou em diferentes territórios. Contudo, o papel do Reino de Portugal nesse processo parece mais tímido, já que a documentação que analisamos aponta para o protagonismo de senhores escravistas e governadores locais.

De qualquer maneira, é inegável que os escravizados não apenas desempenharam papel fundamental na viabilização da chegada da vacina ao Brasil e no seu posterior envio para Angola na primeira década do século XIX, como também tiveram seus corpos utilizados nesse processo, figurando entre os primeiros vacinados nos domínios portugueses do ultramar e sendo instrumentalizados em redes de disseminação do método preventivo nesses territórios.

Ao analisarmos as medidas tomadas para o combate de uma doença letal como a varíola, que foi responsável por inúmeras epidemias ao longo da história, revela-se a importância da circulação do conhecimento científico em terras coloniais e a adaptação e elaboração de sujeitos históricos com vistas a “prevenir” os efeitos devastadores das doenças epidêmicas. Desse modo, a temática proposta buscou contemplar a história das ciências em territórios dominados pelo Império português, ao mesmo tempo que recuperou as peculiaridades de sociedades escravistas e hierárquicas.

Conhecer esse cenário contribui para o entendimento das interseções envolvendo saúde, ciência e escravidão no combate às epidemias e na elaboração de soluções para contê-las ao longo da história. Além disso, analisando o caso da vacina antivariólica, observou-se a posição de uma colônia mais protagonista dos conflitos do seu tempo do que passiva e receptora de ideias prontas. Na adaptação das novidades em torno da ciência experimental, entretanto, a escravidão e as relações sociais de dominação constituíram o legado fundamental para a compreensão de aspectos relacionados à chegada e posterior circulação da vacina em espaços controlados pelo Império português em princípios do século XIX.

## Data Availability

Não estão em repositório.

## NOTAS

2 A doença comumente levava ao óbito dos infectados. Entre os sobreviventes, a cegueira e as cicatrizes pelo corpo eram marcas frequentes associadas à passagem da varíola (Gazeta, 2006).

3 Dauril Alden e Joseph Miller (1987, p.195) apresentaram dados sobre as origens e disseminação da varíola no Brasil e apontam o tráfico transatlântico de escravizados como um potencial transmissor de doenças nas Américas. Segundo os autores, a varíola foi um dos principais fatores que provocou mortes na travessia nos navios, ou seja, a população negra foi a mais atingida pela doença.

4 Destaca-se que os povos indígenas também fizeram parte do grupo alvo das práticas de inoculação e vacinação antivariólica no início do século XIX. A vacinação, inicialmente voltada para os escravizados, também se estendeu a outras populações vulneráveis, conforme argumentou Barbosa (2019). Em 1799, o príncipe regente dom João VI emitiu ordem para que “se introduzisse e promovesse a inoculação em crianças negras e índias” (Hochman, Sousa, 2022; Brizola, 2022, p.244).

5 Felisberto Caldeira Brant estava informado dos resultados da expedição espanhola da vacina nas Américas. O experimento dos brasileiros, com o apoio da Coroa portuguesa, guarda semelhanças com o método adotado pelos médicos Francisco Javier de Balmis e José Salvani, que, em 1803, partiram de La Coruña, na Espanha com 22 meninos carregando o material varioloso em seus corpos (Ramírez Martín, 2002). A Real Expedição Filantrópica da Vacina é considerada a primeira e mais importante tentativa de vacinação em massa da história, já que alcançou praticamente toda a porção sul do continente americano, com exceção do Brasil, e contemplou localidades na África e na Ásia (Balaguer I Periguell, Ballester, 2003; Ramírez Martín, 2002; Tuells, Duro Torrijos, 2015).

6 Para detalhes sobre a introdução da vacina antivariólica em Portugal, consultar Barreiros (2014, p.218) e Portugal (2018, p.42).

7 Segundo Renato Tourinho Dantas (out. 1978), José Avelino Barbosa, o primeiro vacinador da Bahia, pertencia a um seleto grupo de médicos brasileiros com formação acadêmica no exterior. Graduou-se em medicina, na Universidade de Edimburgo. Em 1815, passou a ministrar aulas no Colégio Médico Cirúrgico na Bahia. Além disso, participou ativamente da vida social e política na época da independência do Brasil e foi eleito deputado provincial pela Bahia em 1826 (Oliveira, 1992, p.34).

8 A expressão “trocas sanitárias”, cunhada por Maria Gabriela Marinho (2015, p.156, 157), refere-se às informações que circulavam entre as capitanias e a Corte em termos de “saúde” em princípios do século XIX, assim como às trocas dos próprios governadores e suas respectivas experiências com práticas de inoculação do pus variólico. Tendo em conta essa ampla circulação, captável pela análise de documentos presentes em diferentes arquivos, a autora identifica um viés especificamente sanitário na administração colonial.
